# Combined germline and tumor mutation signature testing identifies new families with *NTHL1* tumor syndrome

**DOI:** 10.3389/fgene.2023.1254908

**Published:** 2023-08-31

**Authors:** Carla Pinto, Joana Guerra, Manuela Pinheiro, Carla Escudeiro, Catarina Santos, Pedro Pinto, Miguel Porto, Carla Bartosch, João Silva, Ana Peixoto, Manuel R. Teixeira

**Affiliations:** ^1^ Department of Laboratory Genetics, Portuguese Oncology Institute of Porto (IPO-Porto)/Porto Comprehensive Cancer Center, Porto, Portugal; ^2^ Cancer Genetics Group, IPO-Porto Research Center (CI-IPOP)/RISE@CI-IPOP (Health Research Network), Portuguese Oncology Institute of Porto (IPO-Porto)/Porto Comprehensive Cancer Center, Porto, Portugal; ^3^ Department of Pathological, Cytological and Thanatological Anatomy, School of Health, Polytechnic Institute of Porto, Porto, Portugal; ^4^ Doctoral Programme in Biomedical Sciences, School of Medicine and Biomedical Sciences (ICBAS), University of Porto, Porto, Portugal; ^5^ Department of Pathology, Portuguese Oncology Institute of Porto (IPO-Porto)/Porto Comprehensive Cancer Center, Porto, Portugal; ^6^ Cancer Biology and Epigenetics Group, IPO-Porto Research Center (CI-IPOP)/RISE@CI-IPOP (Health Research Network), Portuguese Oncology Institute of Porto (IPO-Porto)/Porto Comprehensive Cancer Center, Porto, Portugal; ^7^ Department of Medical Genetics, Portuguese Oncology Institute of Porto (IPO-Porto)/Porto Comprehensive Cancer Center, Porto, Portugal; ^8^ School of Medicine and Biomedical Sciences (ICBAS), University of Porto, Porto, Portugal

**Keywords:** *NTHL1*, polyposis, colorectal cancer, multi-tumor syndrome, recessive disorder

## Abstract

*NTHL1* tumor syndrome is an autosomal recessive rare disease caused by biallelic inactivating variants in the *NTHL1* gene and which presents a broad tumor spectrum. To contribute to the characterization of the phenotype of this syndrome, we studied 467 index patients by KASP assay or next-generation sequencing, including 228 patients with colorectal polyposis and 239 patients with familial/personal history of multiple tumors (excluding multiple breast/ovarian/polyposis). Three *NTHL1* tumor syndrome families were identified in the group of patients with polyposis and none in patients with familial/personal history of multiple tumors. Altogether, we identified nine affected patients with polyposis (two of them diagnosed after initiating colorectal cancer surveillance) with biallelic pathogenic or likely pathogenic *NTHL1* variants, as well as two index patients with one pathogenic or likely pathogenic *NTHL1* variant in concomitance with a missense variant of uncertain significance. Here we identified a novel inframe deletion classified as likely pathogenic using the ACMG criteria, supported also by tumor mutational signature analysis. Our findings indicate that the *NTHL1* tumor syndrome is a multi-tumor syndrome strongly associated with polyposis and not with multiple tumors without polyposis.

## 1 Introduction

*NTHL1* tumor syndrome, also known in the literature as *NTHL1*-associated polyposis, is a multi-tumor spectrum disease, whose narrow spectrum is mostly characterized by an increased lifetime risk for colorectal polyposis, colorectal cancer (CRC), and breast cancer (BC) (Kuiper et al., 2020). This syndrome is caused by biallelic inactivating variants in the *NTHL1* gene (NM_002528.7) and is inherited in an autosomal recessive manner. The *NTHL1* gene encodes a DNA glycosylase that specifically targets oxidized pyrimidine residues in DNA and has an apurinic/apyrimidinic lyase activity that plays an important role in the Base Excision Repair (BER) mechanism (Robertson et al., 2009; Wallace et al., 2012). Consequently, biallelic inactivation of this gene deeply compromises DNA repair mediated by the BER pathway, leading to a gradual accumulation of somatic variants (mostly C>T transitions) that increases the risk of cancer in tissues more susceptible to this type of damage (Weren et al., 2015).

This syndrome was described for the first time in 2015 by Weren and others, who reported three Dutch families with the same homozygous truncating variant in the *NTHL1* gene, which was reported at the time as c.268C>T p.(Gln90Ter) using the NM_002528.6, (Weren et al., 2015), but that is currently described as c.244C>T p.(Gln82Ter) according to NM_002528.7, with ClinVar ID 192319. Weren and co-workers hypothesized that this may be a founder Dutch variant, but, meanwhile, several families were described worldwide harboring the same homozygous variant, or in compound heterozygosity with other truncating variants in the same gene (Grolleman et al., 2019; Fostira et al., 2018; Belhadj et al., 2017; Chubb et al., 2016; Rivera et al., 2015; Weren et al., 2015; Altaraihi et al., 2019; Boulouard et al., 2021). Additionally, other loss of function variants in *NTHL1* have been described in the literature (Grolleman et al., 2019; Kuiper et al., 2020; Boulouard et al., 2021). Tumors of the biallelic *NTHL1* variant carriers exhibit a distinctive COSMIC somatic mutation pattern, the single base substitution signature 30 (SBS30), characterized by an abundance of C>T transitions predominantly at non-CpG sites (Grolleman et al., 2019).

Although inactivation of the *NTHL1* gene was initially described as being associated with polyposis and CRC, the spectrum of neoplasms presented by the families is widening (Grolleman et al., 2019; Kuiper et al., 2020). In fact, other malignant and non-malignant lesions may occur in the broad spectrum of this syndrome, namely, endometrial malignancies and premalignancies, cervical cancer, urothelial carcinoma of the bladder, meningiomas, brain tumors, basal cell carcinomas, head and neck squamous cell carcinomas, and hematologic malignancies (Kuiper et al., 2020). Given the scarce number of families identified so far, a correct characterization of the cancer spectrum is difficult and diagnosis of this syndrome can easily be missed. Therefore, cancer risk estimates have not been well established, making the genetic counseling process challenging. In this study, we tested two consecutive series of patients, one with colorectal polyposis and the other with familial/personal history of multiple tumors (besides multiple breast/ovarian/polyposis), in order to contribute to characterization of *NTHL1* tumor syndrome associated phenotype.

## 2 Materials and methods

### 2.1 Patients and samples

A total of 467 index patients referred to the Genetics Department of the Portuguese Oncology Institute of Porto (IPO-Porto) were included in this study, 412 patients were selected retrospectively, and 55 patients were prospectively enrolled. The 467 index patients were divided in two groups. The first group comprised 228 patients with colorectal polyposis that were negative for *MUTYH* and/or *APC* pathogenic variants, in whom the entire coding regions and intronic adjacent regions of *MUTYH* and/or *APC* genes had been studied (149 patients) or the most frequent *MUTYH* variants in our population (the NM_001128425.1:c.536A>G p.(Tyr179Cys), the NM_001128425.1:c.1187G>A p.(Gly396Asp), and the NM_001128425.1:c.1227_1228dup p.(Glu410GlyfsTer43)) had been screened (79 patients). In the second group we included 239 patients with familial/personal history of multiple tumors (except multiple breast, ovarian tumors or polyposis) without pathogenic variants in the high penetrance genes associated with the corresponding phenotypes (according to the geneticist evaluation). This study was approved by the Ethics Committee of IPO-Porto (reference number CES 112/021) and was conducted according to the Helsinki Declaration. Informed consent for research was obtained from all the patients at the time of genetic counselling. Whenever possible, family members of the positive index patients were also studied. Genomic DNA was extracted from peripheral blood samples according to standard protocols.

### 2.2 *NTHL1* variant screening

All *NTHL1* variants were described according to the Human Genome Variation Society (HGVS) nomenclature and using NM_002528.7 as reference sequence.

#### 2.2.1 Kompetitive allele-specific PCR genotyping assay

In all patients selected retrospectively or negative for the most frequent *MUTYH* variants selected prospectively (*n* = 429; 193 with polyposis and 236 with multiple tumors), the recurrent *NTHL1* variant NM_002528.7:c.244C>T p.(Gln82Ter) was screened using a kompetitive allele-specific PCR (KASP) genotyping assay (LGC Genomics, Hoddesdon, UK) according to the manufacturer´s protocol in a LightCycler^®^ 480 Real-Time PCR System (Roche, Basel, Switzerland). The primers were designed using the Primer-BLAST design tool from NCBI (Ye et al., 2012).

#### 2.2.2 Next-generation sequencing

In 2019, the *NTHL1* gene was included in the updated version of the TruSight Hereditary Cancer panel (Illumina, Inc., San Diego, CA, United States). Since then, 38 patients fulfilled the criteria to be included in this study: 35 patients presented polyposis and three patients had familial/personal history of multiple tumors. Briefly, the *NTHL1* gene was analyzed by NGS, using the aforementioned kit and the Illumina DNA Prep with Enrichment protocol (Illumina, Inc.), with library preparation performed according to the manufacturer’s protocol, and the sequencing reaction carried out using a high-output kit in the NextSeq 550 platform (Illumina, Inc.) in 2x150 bp paired end runs. Sequencing alignment and variant analysis were performed using a bioinformatics pipeline previously validated by our group (Paulo et al., 2017). In brief, alignment and variant calling were done using NextGENe (v2.4, Softgenetics, State College, PA, United States), with.vcf files being imported into GeneticistAssistant™ (Softgenetics) for variant annotation. All variants with a variant allele frequency (VAF) ≤10%, minor allele frequency (MAF) > 1%, in-house database frequency ≥5%, and/or intronic variants at more than 12bp away from exon-intron boundaries were excluded. For MAF filtering, data was obtained from the 1000 Genomes Project (1KGP), Genome Aggregation Database (gnomAD), and Exome Aggregation Consortium (ExAC) databases.

#### 2.2.3 Sanger sequencing

Sanger sequencing was used to validate the KASP genotyping results, to sequence the entire *NTHL1* coding region whenever a heterozygous carrier was identified during the KASP genotyping screening, and to study all family members available. Briefly, Sanger sequencing was performed using the BigDye^®^ Terminator v3.1 Cycle Sequencing Kit (ThermoFisher Scientific, Waltham, MA, United States), following the manufacturer’s instructions, and the products were analyzed on an ABI 3500 Genetic Analyzer (ThermoFisher Scientific).

### 2.3 Comprehensive tumor genomic profiling and mutational signature

DNA extraction from FFPE samples of three different *NTHL1* variant carriers (patient #1 and #7, and relative #3) was performed using the Cobas^®^ DNA Sample Preparation Kit (Roche Diagnostics, Basel, Switzerland) according to the manufacturer’s protocol. Quantity of DNA samples was measured with the Qubit dsDNA HS assay kit in a Qubit^®^ Fluorometer (Thermo Fisher Scientific). DNA libraries were prepared using the Illumina TruSight Oncology 500 (TSO500) kit (Illumina, Inc.) according to the manufacturer’s instructions. Sequencing was carried out using a high-output kit in the NextSeq 550 System (Illumina, Inc) in a 2x101 bp paired-end run.

Data analysis was performed using the TSO500 Local App v2.0 (Illumina, Inc) and the resulting.vcf files were imported to GeneticistAssistant™ software (SoftGenetics) for variant annotation. The following variant calls were excluded: variants with a MAF of >0.01% in GnomAD, variants in non-exonic regions, and/or insertions and deletions with in-house database frequency ≥20%.

To infer the contribution of the Catalogue of Somatic Mutations in Cancer (COSMIC) mutational signatures (v3.2), we used the MutationalPatterns package in R v4.2.1 (Manders et al., 2022). This analysis was also applied to a set of eight sporadic endometrial cancers and 18 colorectal tumors.

## 3 Results

### 3.1 *NTHL1* germline variants

A total of 467 patients were studied, 228 with colorectal polyposis and 239 with familial/personal history of multiple tumors. Seven *NTHL1* biallelic variant carriers were identified in index patients with polyposis and no biallelic *NTHL1* variants were identified in patients with familial/personal history of multiple tumors (Table 1). Of the *NTHL1* biallelic variant carriers, four patients harbor the variant NM_002528.7:c.244C>T p.(Gln82Ter) in homozygosity, three identified by the KASP assay and one identified by NGS.

**TABLE 1:** Phenotypic characteristics of the *NTHL1* biallelic variant carriers.

Family	*NTHL1* variant		Patient	Gender	Phenotype [age at diagnosis]
	DNA (NM_002528.7)	Predictive protein			
1	c.[244C>T];[244C>T]	p.[(Gln82Ter)];[(Gln82Ter)]	^a^Patient #1	F	CRC [58], polyposis [62], BC [66], endometrial polyps
2	c.[244C>T];[244C>T]	p.[(Gln82Ter)];[(Gln82Ter)]	^a^Patient #2	M	Polyposis [47], PaC [47]
			^a^Patient #3	M	Polyposis, CRC [47]
			^a^Patient #4	M	Polyposis, CRC [49]
			Relative #1	F	Healthy (23 polyps after screening)
			Relative #2	M	Healthy (4 polyps after screening)
3	c.[244C>T];[435_446del]	p.[(Gln82Ter)];[(Leu146_Arg149del)]	^a^Patient #5	F	Polyposis, rectal cancer [52], BC [54, 58], uterine carcinosarcoma [58]
			Relative #3	M	CRC [62], CRC [62], polyposis [62]
			Relative #4	M	Polyposis [50], basal cell carcinoma
4	c.244C>T(;)(503T>C)	p.(Gln82Ter)(;)(Ile168Thr)	^a^Patient #6	M	Polyposis [68]
5	c.[115+1G>A];[503T>C]	p.[?];[(Ile168Thr)]	^a^Patient #7	F	EC [66], uterine leiomyosarcoma [66] (2 polyps after screening)

^a^ Index patient. BC, breast cancer; EC, endometrial cancer; CRC, colorectal cancer; PaC, pancreatic cancer; F, Female; M, male.

**FIGURE 1:**
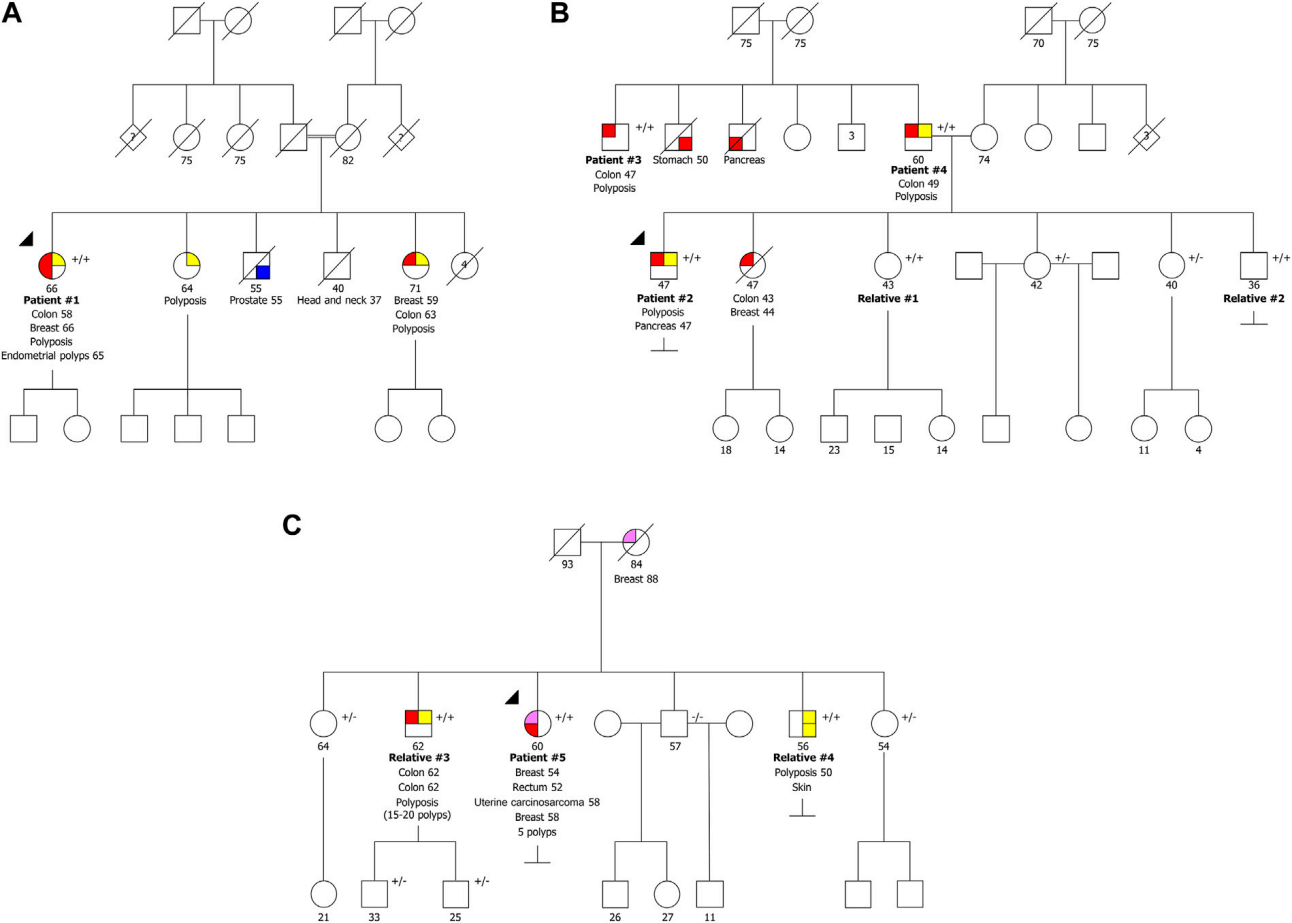
Pedigrees of the three families with biallelic pathogenic variants in *NTHL1* gene. **(A)** Family presenting the variants NM_002528.7:c.[244C>T]; [244C>T] p.[(Gln82Ter)];[(Gln82Ter)]. **(B)** Family presenting the variants NM_002528.7:c.[244C>T];[244C>T] p.[(Gln82Ter)];[(Gln82Ter)]. **(C)** Family presenting the variants NM_002528.7:c.[244C>T];[435_446del] p.[(Gln82Ter)];[(Leu146_Arg149del)]. +/+ biallelic carrier, +/− heterozygous carrier, −/− wild type.

Patient #1 is a woman presenting the variant NM_002528.7:c.244C>T p.(Gln82Ter) in homozygosity, who was diagnosed with CRC at 58 years old, with at least 24 adenomatous and eight hyperplastic polyps at 62 years old, endometrial polyps, and BC at the age of 66. One of her sisters had polyposis, BC at 59 and CRC at 63 years old, and other sister had polyposis at 64 years old, but none of them have been tested yet (Figure 1A). She had two deceased brothers, one of them diagnosed with prostate cancer at age 55 and the other presented head and neck cancer at 37 years old. Additionally, she also had four deceased sisters that, to our knowledge, did not develop cancer. According to the information mentioned by the index patient during genetic counseling, the parents are consanguineous, but she was not sure exactly how they were related.

Patient #2 is a man also presenting the variant NM_002528.7:c.244C>T p.(Gln82Ter) in homozygosity, who had been diagnosed with polyposis (less than 100 polyps) and pancreatic cancer at 47 years old. His deceased sister had CRC and BC at age 43 and 44, respectively (Figure 1B). We were able to test the other four healthy siblings, and two are homozygous (relatives #1 and #2) and two are heterozygous carriers. The healthy relatives presenting the variant in homozygosity have already initiated CRC surveillance and after colonoscopy both presented colorectal polyps (relative #1 had 23 polyps and relative #2 had four polyps). Additionally, we identified two homozygous carriers in the retrospectively series, two men (patients #3 and #4), apparently not related, diagnosed with polyposis (both with more than 100 polyps) and CRC, at 47 and 49 years old, respectively, with no information available regarding family history. Unexpectedly, we found out later that these two men were father and uncle of patient #2 (Figure 1B), who had strong family history of colon, stomach and pancreatic cancer of the paternal side not expected in a disease with an autosomal recessive inheritance.

Patient #5 was a compound heterozygous carrier presenting the recurrent variant NM_002528.7:c.244C>T p.(Gln82Ter) in concomitance with the NM_002528.7:c.435_446del p.(Leu146_Arg149del) variant. This patient was a woman diagnosed with five polyps and rectal cancer at 52 years, bilateral BC at 54 and 58 years and uterine carcinosarcoma at 58 years. She had two brothers with biallelic variants (relatives #3 and #4), one diagnosed with two synchronous CRCs and polyposis (15-20 polyps) at 62 years old and another diagnosed with polyposis at 50 years old and a basal cell carcinoma (Figure 1C). Furthermore, she had three healthy siblings, two carriers of the inframe variant and one negative for both variants. Relative #3 has two healthy sons presenting only the inframe variant. The fact that there are relatives harboring only one of the variants confirms the biallelic nature of these variants. The variant NM_002528.7:c.435_446del, p.(Leu146_Arg149del), has not previously been described in the literature. Using the ACMG classification, this variant is classified as likely pathogenic as it is absent in population databases (no frequency in gnomAD) (PM2), co-segregates with the disease in this family (PP1), the protein length changes as a result of an inframe deletion in a nonrepeat region (PM4), and it was detected in *trans* with a pathogenic variant in a recessive disorder (PM3).

**FIGURE 2:**
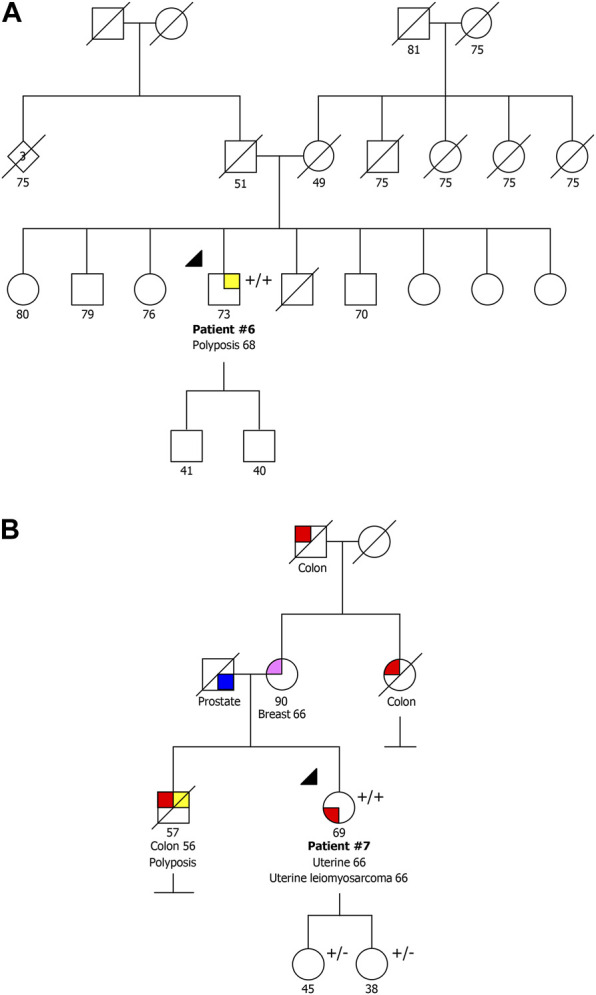
Pedigrees of the two families with a pathogenic/likely pathogenic variant co-occurring with the VUS NM_002528.7:c.503T>C p.(Ile168Thr). **(A)** Family also presenting the variant NM_002528.7:c.244C>T p.(Gln82Ter). **(B)** Family also presenting the variant NM_002528.7:c.115+1G>A. +/+ biallelic carrier, +/− heterozygous carrier.

Patient #6 was a compound heterozygous carrier presenting the recurrent NM_002528.7:c.244C>T p.(Gln82Ter) variant and the NM_002528.7:c.503T>C p.(Ile168Thr) variant. This patient was a man diagnosed with oligopolyposis (<10) at age 68 without cancer family history (Figure 2A). The variant NM_002528.7:c.503T>C p.(Ile168Thr) has previously been described as heterozygous in the literature in individuals with attenuated adenomatous polyposis, CRC and ovarian cancer (Belhadj et al., 2019; Lorca et al., 2019; Shickh et al., 2021). It has also been reported as homozygous by Boulouard and collaborators in two individuals with breast, prostate and endometrial cancer (Boulouard et al., 2021), and has a conflicting classification (Uncertain significance/Likely benign) in ClinVar (ID 587357). Using the ACMG classification, this variant is classified as variant of uncertain significance (VUS), as it is present in population databases (0.00083 in gnomAD, including one homozygous) (BS2), it was detected in *trans* with a pathogenic variant in a recessive disorder (PM3), and it is a missense variant for which *in silico* tools suggest a deleterious effect on protein function, though these predictions have not been confirmed by functional studies (PP3).

Patient #7 was a compound heterozygous carrier presenting the same variant detected in patient #6, NM_002528.7:c.503T>C p.(Ile168Thr), in association with the NM_002528.7:c.115+1G>A variant. This patient was a woman diagnosed with synchronous endometrial carcinoma and uterine leiomyosarcoma at 66 years of age. Although this patient belonged to the group of patients with familial/personal history of multiple tumors, after the genetic test result she initiated colonoscopy screening and two polyps were detected. She had a deceased brother diagnosed with CRC at 56 years and polyposis, and family history of BC and CRC with unknown age of onset (Figure 2B). Additionally, both healthy daughters were studied, and were heterozygous carriers, which confirms the biallelic status of these variants. The variant NM_002528.7:c.115+1G>A has been described as pathogenic/likely pathogenic in ClinVar (ID 656884) and affects a donor splice site expecting to cause aberrant splicing.

Furthermore, two heterozygous carriers were identified, one with the variant NM_002528.7:c.244C>T p.(Gln82Ter) and another with the variant NM_002528.7:c.526-1G>A. The first heterozygous carrier fulfilled the criteria of familial/personal history of multiple tumors, and the latter was a patient presenting polyposis.

Therefore, in this study we identified nine patients affected with polyposis (two of them diagnosed after initiating CRC surveillance) with biallelic pathogenic or likely pathogenic *NTHL1* variants belonging to three separate families (Figure 1), as well as two families with one pathogenic or likely pathogenic *NTHL1* variant in concomitance with the VUS NM_002528.7:c.503T>C p.(Ile168Thr) (Figure 2).

### 3.2 Somatic mutational signatures

**FIGURE 3:**
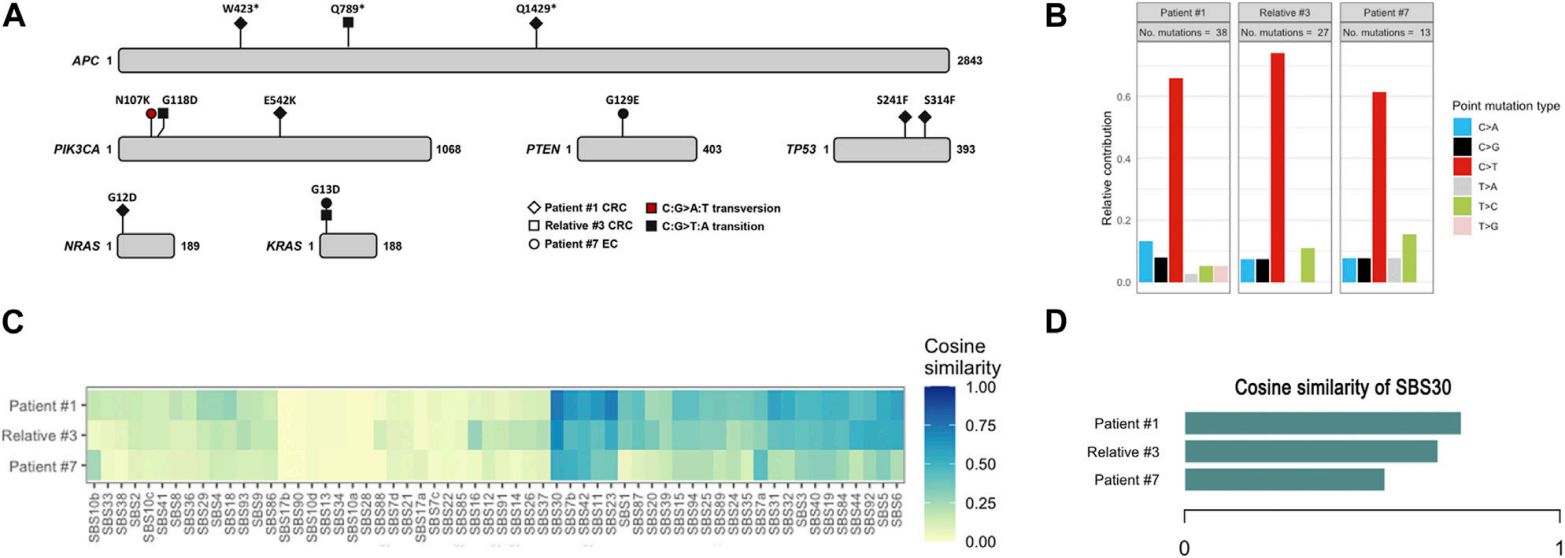
Somatic mutation data of colorectal cancers of patient #1 and relative #3, and of endometrial cancer of patient #7. **(A)** Spectrum of mutations detected in driver cancer genes. **(B)** Relative contribution of C>T transitions. **(C)** Heatmap depicting the cosine similarity with the COSMIC signatures. **(D)** Cosine similarity scores of SBS30. EC, endometrial cancer; CRC, colorectal cancer.

To better evaluate the biological impact of the novel inframe deletion and of the recurrent missense variant, we performed a comprehensive genomic characterization and determination of somatic mutational signatures of three tumors. We analyzed the CRC of patient #1 with the known recurrent variant in homozygosity, the CRC of relative #3 (from family #3) with the novel inframe variant (concomitant with the recurrent variant), and the endometrial cancer of patient #7 carrier of a pathogenic variant and a missense variant. We detected somatic variants in several known cancer driver genes, such as *APC, PIK3CA*, *TP53*, *PTEN, KRAS* and *NRAS* (Figure 3A). Most somatic mutations were C>T transitions (Figure 3B), predominantly located at non-CpG sites. Moreover, we assessed the contribution of the COSMIC mutational signatures, and the SBS30 was observed predominantly in all three tumors, but with less representativity in the endometrial cancer of patient #7. The relative contribution of SBS30 was 0.29, 0.37 and 0.19 for tumors from patient #1, relative #3 and patient #7, respectively, and only two tumors from the control set presented a residual contribution of the SBS30 (Supplementary Table S1). The cosine similarities values of the reconstructed mutation profile were 0.74 for CRC of patient #1, 0.68 for CRC of relative #3, and 0.53 for the endometrial cancer of patient #7 (Figures 3C, D). Additionally, all three tumors were microsatellite stable (MSS) and tumor mutation burden values were 12.7, 20.1, and 3.9 for tumors from patient #1, relative #3 and patient #7, respectively.

## 4 Discussion

As only few families with *NTHL1* tumor syndrome had been reported in the literature, the mutation pattern in different populations is still poorly established. Our study describes three new *NTHL1* tumor syndrome families, two of them presenting in homozygosity the recurrent pathogenic variant NM_002528.7:c.244C>T p.(Gln82Ter), formerly known as c.268C>T, p.(Gln90Ter), using the NM_002528.6, and one family harboring that variant in compound heterozygosity with the variant NM_002528.7:c.435_446del p.(Leu146_Arg149del) (Figure 1). The latter variant has not previously been described in the literature and it is here classified as likely pathogenic following the ACMG guidelines, a classification that is also supported by the evidence from the tumor mutational signature analysis we here present.

In the three families described in our study, we were able to identify nine pathogenic *NTHL1* biallelic variant carriers (homozygous or compound heterozygous), of which seven were patients that had polyposis diagnosed before genetic testing and two were relatives that, after a positive pre-symptomatic genetic testing, presented colorectal polyps in the first colonoscopies. Of the five affected male biallelic variant carriers, in addition to polyposis, three presented CRC (one of them with two synchronous CRCs), one had basal cell carcinoma, and another presented pancreatic cancer. Both affected female biallelic variant carriers presented, in addition to polyposis, CRC, BC (one of them bilateral), and uterine lesions (Table 1). In our group of pathogenic *NTHL1* biallelic variant carriers, it is evident that, as described in the literature, polyposis, CRC and BC are the main phenotype characteristics of this syndrome (Kuiper et al., 2020; Grolleman et al., 2019). Although we excluded patients with multiple breast/ovarian cancers without polyposis from this study, routine testing since 2019 in our department did not find any such patient with biallelic *NTHL1* deleterious variants, although there are cases reported in the literature presenting with bilateral breast cancer before the colorectal phenotypes (Weatherill et al., 2023). In addition to polyposis, CRC and BC, we found uterine lesions, basal cell carcinoma and pancreatic cancer in our *NTHL1* tumor syndrome families. Uterine lesions and basal cell carcinoma were previously reported in several patients, however, to our knowledge, pancreatic cancer was only described before in one male patient with multiple tumors in one of the first families reported (Weren et al., 2015).

In addition, we also identified two families with one pathogenic or likely pathogenic *NTHL1* variant in concomitance with the missense VUS NM_002528.7:c.503T>C p.(Ile168Thr) (Figure 2). Patient #6 was diagnosed with polyposis and had no family history, and patient #7 presented endometrial carcinoma and a uterine lesion (and two polyps after initiating CRC surveillance) and had family history of CRC, BC and polyposis. A specific COSMIC mutational signature, the SBS30, was previously identified in *NTHL1* associated tumors (Grolleman et al., 2019). We assessed the contribution of signature 30 in three available tumors, the CRCs of patient #1 and relative #3, and the endometrial cancer of patient #7. The SBS30 was observed predominantly in all three tumors, but with less representativity in the endometrial cancer of patient #7 (the missense VUS carrier). In addition, to support these results we also assessed the contribution of this signature to the mutation spectrum of sporadic endometrial (*n* = 8) and colorectal (*n* = 18) cancers previously analyzed with same panel in our laboratory and we did not find any meaningful contribution of this signature in these tumors. Although the contribution of the SBS30 in endometrial cancer of patient #7 is somewhat lower compared to the CRCs of the pathogenic *NTHL1* biallelic variant carriers (Figure 3), it remains the predominant observed signature in this tumor in spite of being evaluated in a different tumor type. Given the suggestive somatic mutation signature and the compatible phenotypes observed in both families, it is possible that this missense variant is deleterious, even if hypomorphic as both carriers showed oligopolyposis, but at this time the evidence is not sufficient to re-classify this variant of uncertain clinical significance. To notice that the tumor from this patient presented a low TMB, which is not expected in tumors with a DNA repair deficiency.

The disease associated with biallelic *NTHL1* deleterious variants was initially named *NTHL1*-associated polyposis, but later the name *NTHL1* tumor syndrome was proposed to accommodate for the increased risk for several cancers. Although our study confirms that biallelic *NTHL1* pathogenic variant carriers have a multi-tumor phenotype syndrome, we saw no evidence that the association of multiple cancers, including CRC and BC, in the absence of polyposis, should prompt genetic testing of *NTHL1*, since none of the 239 patients selected using this criterion presented *NTHL1* pathogenic biallelic variants. This has also been reported by other authors (Belhadj et al., 2019), emphasizing that the presence of polyposis remains the main characteristic associated with biallelic *NTHL1* variant carriers. Nevertheless, due to its multi-tumor broad spectrum phenotype, some *NTHL1* families can resemble other hereditary syndromes and may mislead genetic counseling, especially if the clinical information is incomplete. All index patients with personal/family history of polyposis and/or multi-tumor phenotype should ideally be studied using a NGS panel of cancer predisposition genes that includes *NTHL1*.

The risk and penetrance estimates are difficult to obtain due to the scarce number of biallelic *NTHL1* variant carriers reported in the literature. Furthermore, the surveillance of these patients/deleterious variant carriers is very challenging due to the broad phenotype associated with this syndrome. The National Comprehensive Cancer Network (NCCN) guidelines recommends CRC, endometrial cancer and BC surveillance (NCCN Guidelines, 2022). These guidelines are expected to evolve and all surveillance procedures should be adapted to family history due to the extensive tumor spectrum of this syndrome. On the other hand, we identified two index patients and several relatives from *NTHL1* tumor syndrome families who are heterozygous for pathogenic variants in the *NTHL1* gene. The contribution of the heterozygous variants in this gene to the risk of developing cancer or non-malignant diseases is still unclear. *NTHL1* heterozygotes do not appear to be at clinically significant increased risk for polyposis and/or CRC (Elsayed et al., 2020; NCCN Guidelines, 2022) and BC (Kumpula et al., 2020), but a slightly increased risk cannot be excluded (Li et al., 2021), as has been described for patients with heterozygous variants in the *MUTYH* gene (Win et al., 2014).

In conclusion, this study used the mutational signature previously identified in *NTHL1* associated tumors as compared with sporadic tumors, which was instrumental to classify as likely pathogenic a novel inframe deletion, but that it was not sufficient to classify the recurrent missense variant as anything else than a variant of uncertain clinical significance. Our findings further indicate that the *NTHL1* tumor syndrome is a multi-tumor syndrome strongly associated with polyposis and not with multiple tumors without polyposis.

## Data Availability

The data presented in the study are deposited in the https://www.ebi.ac.uk/ena repository, accession number PRJEB65306.
