# Supplementary material for: Combined germline and tumor mutation signature testing identifies new families with NTHL1 tumor syndrome
Source: Front Genet. 2023 Aug 31;14:1254908. doi: 10.3389/fgene.2023.1254908 (PMC10505957; doi:10.3389/fgene.2023.1254908)
Supplement: Supplementary file 1 [file Table1.DOCX]

**Supplementary table 1** – The estimated relative contribution of SBS30 to the mutation spectrum of tumors from patient #1, relative #3, patient #7, and sporadic tumor controls.

|  | Patient #7 | Relative #3 | Patient #1 | CC1 | CC2 | CC3 | CC4 | CC5 | CC6 | CC7 | CC8 | CC9 | CC10 | CC11 | CC12 | CC13 | CC14 | CC15 | CC16 | CC17 | CC18 | CE1 | CE2 | CE3 | CE4 | CE5 | CE6 | CE7 | CE8 |
| --- | --- | --- | --- | --- | --- | --- | --- | --- | --- | --- | --- | --- | --- | --- | --- | --- | --- | --- | --- | --- | --- | --- | --- | --- | --- | --- | --- | --- | --- |
| SBS30 | 0.19 | 0.37 | 0.29 | 0 | 0 | 0 | 0 | 0 | 0 | 0 | 0 | 0 | 0 | 0 | 0 | 0 | 0 | 0.029 | 0 | 0 | 0 | 0 | 0 | 0 | 0 | 0 | 0 | 0.069 | 0 |

CC, control colon cancer; CE, control endometrial cancer, SBS, single base substitution.
